# Panic Disorder Induced by the Coronavirus Disease Pandemic in a Patient with Organic Mood Disorder Successfully Treated with Vortioxetine

**DOI:** 10.1155/2020/8870014

**Published:** 2020-11-02

**Authors:** Reiji Yoshimura, Naomichi Okamoto, Yuki Konishi, Atsuko Ikenouchi

**Affiliations:** Department of Psychiatry, University of Occupational and Environmental Health, Japan, 1-1 Iseigaoka, Yahatanishi-ku, Kitakyushu, Fukuoka 8078555, Japan

## Abstract

We present a case of panic disorder induced by the coronavirus disease (COVID-19) pandemic in a patient with an organic mood disorder. The patient was a 62-year-old woman with mild mood swings and reduced motivation and volition caused by a traumatic brain injury after a traffic accident. She was maintained on carbamazepine (600 mg/day). When the COVID-19 outbreak occurred in Japan, she developed concerns regarding the illness and complained of multiple episodes of panic attacks. Further, her depressive symptoms worsened. Thus, vortioxetine was added to the ongoing CBZ treatment. Four weeks after initiating vortioxetine treatment, the symptoms of panic disorder and depressive state were ameliorated. The present case suggests that vortioxetine augmentation can improve symptoms of depressive state and panic disorder induced by the COVID-19 pandemic.

## 1. Introduction

The pandemic of the novel coronavirus disease (COVID-19) has caused concern regarding the mental health of the healthy population as well as patients with psychiatric disorders [1]. Therefore, alternative management strategies that encompass the needs of psychiatric patients should be considered. Here, we present a case report of a patient with an organic mood disorder due to a traumatic brain injury caused by a traffic accident, who developed a COVID-19-related panic disorder comorbid with an organic mood disorder.

## 2. Case Presentation

A 62-year-old woman was initially presented as an outpatient ten years ago to the Department of Psychiatry at the University Hospital of Occupational and Environmental Health in Japan. She presented with mild mood swings, impulsive aggression, and reduced motivation and volition caused by a traumatic brain injury after a traffic accident. She had no previous history of substance abuse, hypertension, diabetes mellitus, or thyroid dysfunction. Magnetic resonance imaging revealed small hyperintensities in the left temporal and anterior frontal lobes, hippocampus, and amygdala. Based on the Wechsler Adult Intelligence Scale-Third Edition, the verbal intelligence quotient (IQ), performance IQ, and full IQ were 91, 96, and 92, respectively, which indicated normal intelligence. Her Mini-Mental State Examination score was 30/30, which indicated no cognitive impairment. She was often emotionally unstable; further, she was occasionally aggressive and cried after quarreling with her family members.

We initiated carbamazepine (CBZ) at an initial dose of 200 mg/day, which was increased to 600 mg/day. After four weeks, her mood swings gradually improved. She remained stable taking CBZ; however, she continued to present reduced motivation and volition. When the COVID-19 outbreak occurred in Japan, social media became inundated with COVID-19-related content. Consequently, she became concerned about COVID-19 and complained of multiple episodes of severe anxiety and shortness of breath that involved excessive sweating, body trembling, mouth dryness, and fear of death in public spaces. As a result, she could not go to the supermarket by herself due to excessive fear of contracting the illness. Further, her depressive mood worsened. Based on these symptoms, she was diagnosed with panic disorder comorbid with organic mood disorder and was prescribed sertraline (25 mg/day, which was increased to 100 mg/day) and 0.4 mg alprazolam as required. Her panic symptoms did not improve; consequently, vortioxetine was started and increased to 20 mg/day, while sertraline was tapered off. Four weeks after initiating vortioxetine, her symptoms of panic disorder and depressive mood, and reduced motivation were ameliorated. Further, her plasma level of CBZ was 6.4 *μ*g/ml. Although the COVID-19 pandemic continues in Japan, she has remained well without any panic attacks while continuing vortioxetine treatment. The patient has been in remission of all mood symptoms and panic attacks. Written informed consent was obtained from the patient for publication of the present case report.

## 3. Discussion

To the best of our knowledge, this is the first study to report that vortioxetine augmentation can improve depressive symptoms and panic disorder related to the COVID-19 pandemic. The rapid worldwide spread of the pandemic has elicited a considerable degree of fear and anxiety. Experts suggest that these concerns should be monitored in specific populations, including the elderly and individuals with comorbid psychiatric disorders [2]. The patient remained stable with CBZ treatment for a long time. Importantly, CBZ has been previously reported to be effective for the treatment of mood swings, depressive state, and impulsive aggression in organic mood disorder after brain injury [3–5].

The present case suggests that COVID-19-related fear and anxiety may develop into panic disorder. Further, it indicates that clinicians may notice that patients with traumatic brain injuries may present with excessive fear and anxiety during the COVID-19 pandemic, which could be effectively treated with vortioxetine. In the present case, treatment with vortioxetine improved the symptoms of panic disorder and depressive mood. Although the COVID-19 pandemic continues in Japan, the patient has remained well without any occurrences of panic attacks while continuing vortioxetine treatment. Therefore, we considered that the panic disorder occurred after the COVID-10 pandemic and was not simply a deterioration of the organic mood disorder due to the pandemic stressor.

Vortioxetine has been shown to be effective for anxiety disorders, including panic disorder [6, 7]. Vortioxetine has a broad action profile involving both serotonin (5HT) transporter and several 5HT receptors, including 5HT_3A_, 5HT_7_, and 5HT_1D_ receptor antagonists; 5HT_1B_ partial agonist; and 5HT_1A_ agonist [8]. Sertraline and vortioxetine are both SSRIs; thus, both drugs inhibit the serotonin transporter. The difference between the two antidepressants mainly lies in their receptor profiles. Paroxetine, fluvoxamine, and sertraline increase extracellular serotonin levels in the brain. However, only sertraline increases extracellular dopamine concentrations in the brain [9]. On the other hand, vortioxetine decreases the number of bursts per minute in dopamine and noradrenaline neurons [10]. Therefore, it is possible that these differences in pharmacodynamics might be related to the reason why vortioxetine, but not sertraline, was effective for panic disorder in the present case.

Vortioxetine is primarily metabolized by CYP2D6 and CYP2C19 to a lesser extent [11, 12]. For patients with major depressive disorder, a higher vortioxetine dose may be required to be coadministered with a broad CYP450 inducer, including CBZ [13]. In the present case, plasma CBZ levels did not decrease after vortioxetine administration. Administration of vortioxetine alone or with CBZ ameliorated the symptoms of panic disorder and depressive symptoms, including depressive mood, reduced motivation, and volition, which could be attributed to the pharmacological mechanism of vortioxetine. The patient has been remitted for all mood symptoms and panic attacks. We therefore consider that combination treatment with CBZ and vortioxetine was effective in the present case.

In conclusion, we present a case of panic disorder related to the COVID-19 pandemic in a patient with an underlying organic mood disorder caused by a traumatic brain injury. This report showed that adding vortioxetine to ongoing CBZ treatment was effective for treating the panic disorder and depressive symptoms in this patient with an organic brain disorder.
